# External focus instruction using a paper balloon: impact on trunk and lower extremity muscle activity in isometric single-leg stance for healthy males

**DOI:** 10.3389/fspor.2024.1343888

**Published:** 2024-03-14

**Authors:** Koji Murofushi, Tsuyoshi Morito, Hiroshi Akuzawa, Tomoki Oshikawa, Yu Okubo, Koji Kaneoka, Sho Mitomo, Kazuyoshi Yagishita

**Affiliations:** ^1^ Sports Science Center, Tokyo Medical and Dental University (TMDU), Bunkyo-ku, Japan; ^2^ Government of Japan Ministry of Education Culture Sports Science and Technology, Japan Sports Agency, Chiyoda-ku, Japan; ^3^ Faculty of Sport Sciences, Waseda University, Shinjuku-ku, Japan; ^4^ Institute for Human Movement and Medical Sciences, Niigata University of Health and Welfare, Niigata, Japan; ^5^ Center of General Education, Tokyo Keizai University, Kokubunji-shi, Japan; ^6^ Faculty of Health & Medical Care, Saitama Medical University, Saitama, Japan; ^7^ Clinical Center for Sports Medicine and Sports Dentistry, Tokyo Medical and Dental University, Bunkyo-ku, Japan

**Keywords:** trunk muscle activation, external focus instruction, contralateral training, fine-wire electrode analysis, single-leg stance

## Abstract

**Introduction:**

Core stability is crucial for preventing and rehabilitating lumbar spine injuries. An external focus instruction using a paper balloon is an effective way to activate the trunk muscles. However, the degree of trunk and lower extremity muscle activation during single leg stance with external focus instruction using a paper balloon is unknown. This study aimed to investigate the core muscle involving activity in the trunk and lower extremities on both the support and non-support sides with or without using external focus instruction using a paper balloon during isometric single-leg stance.

**Methods:**

Thirteen healthy males aged 20–28 years volunteered to take part in this study and performed a single leg stance task with and without an external focus instruction, pressing their non-supporting foot onto a paper balloon without crushing it. The participant's muscle electrical activity was recorded during the single leg task using surface EMG and intramuscular EMG for six trunk muscles (transversus abdominis, internal oblique, external oblique, rectus abdominis, multifidus, and lumbar erector spinae) and five lower extremity muscles (gluteus maximus, gluteus medius, adductor longus, rectus femoris, and biceps femoris)

**Results:**

Compared to the normal single leg stance, the external focus instruction task using a paper balloon showed significantly increased transversus abdominis (*p* < 0.001, *p* < 0.001), internal oblique (*p* = 0.001, *p* < 0.001), external oblique (*p* = 0.002, *p* = 0.001), rectus abdominal (*p* < 0.001, *p* < 0.001), lumbar multifidus (*p* = 0.001, *p* < 0.001), lumbar erector spinae (*p* < 0.001, *p* = 0.001), adductor longus (*p* < 0.001, *p* < 0.001), rectus femoris (*p* < 0.001, *p* < 0.001), and biceps femoris (*p* < 0.010, *p* < 0.001) muscle activity on the support and non-support sides.

**Conclusion:**

In conclusion, external focus instruction using a paper balloon significantly activates the trunk and lower extremities muscles on both the support and non-support sides. This finding provides insights for designing programs to improve coordination and balance. The benefits extend to diverse individuals, encompassing athletes, tactical professionals, and the general population, mitigating the risk of injury or falls linked to inadequate lower limb balance.

## 1 Introduction

The concept of “core stability” which representing the combination of both trunk and lower extremity muscle's role in balance/stability/coordination. Poor core stability is thought to increase susceptibility to uncontrolled joint displacements across the kinetic chain from the foot to the lumbar spine, appropriate intervention may result in decreased rates of back and lower extremity injury (1). Core stability is predominantly maintained by the dynamic function of muscular elements and there is a clear relationship between trunk muscle activity and lower extremity movement (2). Core stability stabilize lumber vertebra and crucial for preventing and rehabilitating lumbar spine injuries (3–8). Additionally, exercises to enhance core stability are common in sports and rehabilitation, underscoring the crucial role of muscle coactivation for spinal stability, which is crucial for preventing and treating low back injuries (9–13). Therefore, stabilizing the core muscles in different positions is important.

Single-leg balance, which refers to maintaining stability while standing on one leg, is vital for everyday tasks, such as walking, climbing stairs, and carrying objects, for athletes, single-leg stance control is indispensable for dynamic movements, including running, jumping, cutting, and pivoting (14–19).

Single-leg balance training has shown promise in improving balance performance in healthy individuals (20), it has been associated with enhanced postural control (21), improved ankle range of motion, and functional performance in individuals with ankle sprains (22). Monitoring single-leg stability can be valuable for assessing postural stability in different sports groups (23), particularly in young athletes undergoing sports specialization (24). Moreover, several studies have explored muscle activity during single-leg exercises, highlighting the crucial role of trunk and lower extremity muscles in maintaining balance on a single leg (25–28).

Recent investigations on motor skills, including those involving postural stability, have revealed the performance and learning advantages of directing individuals to maintain an external focus of attention, rather than an internal focus (29, 30). The use of an external focus of attention yields various effects, including enhanced force production during activities such as arm curls (31) and improved standing long jump distances (32). Furthermore, Wulf et al. (33) observed that the performance in externally focused jumps was significantly superior to that in internally focused jumps, with a general reduction in electromyography (EMG) activity.

Murofushi et al. introduced a novel isometric method employing an external focus instruction technique with a soft paper balloon (PB), in which the mechanism alters the degree of co-contraction of the antagonistic muscles to avoid crushing the PB (34, 35). Their findings revealed enhanced trunk and lower trapezius muscle activities during isometric chest squeeze and trunk muscle activities in front plank exercises. Further, a novel external focus instruction technique significantly activated the trunk muscles on both the support and non-support sides during side plank exercises (36).

Building on a novel isometric method that involves an external focus instruction technique using a soft PB concept, we conducted external focus as the instruction to not crush the PB with the non-supporting leg during a single-leg stance in this study. This approach aimed to measure muscle activation in the trunk and lower extremity muscles in both support and non-support side of the body simultaneously, contrasting it with that of a normal single-leg stance in the same position. We hypothesized that external focus instruction using a PB would cause more activation in comparison to the normal single-leg stance condition, on the muscles in the trunk and lower extremities on both the support and non-support sides.

## 2 Methods

### 2.1 Participants

Thirteen healthy men participated in the study (mean age, 23 ± 3 years; height, 170.8 cm ± 5.4 cm; body mass, 64.6 ± 9.3 kg). We have chosen exclusion criteria to avoid the impact on muscle activity due to pain, pain history, or surgical history. The exclusion criteria were as follows: (i) individuals with low back pain or neurological findings; (ii) individuals who underwent abdominal or spinal surgery before the study; and (iii) individuals who experienced bodily injury or trauma within three months before the commencement of the study or experienced pain on the testing day. The participants received a comprehensive explanation about the study protocol, and each participant provided written informed consent prior to their participation. This study was approved by the Ethics Committee of Waseda University. Throughout the study, none of the participants withdrew owing to injury, pain, or discomfort, indicating the successful completion of the study without any adverse effects.

### 2.2 Procedures

As part of the study, the participants were required to perform a static single-leg stance whilst pushing on a PB (diameter, 14 cm; weight, 5 g), with either an external focus instruction or just the single-leg stance without any external focus instruction. The PB was selected according to the criteria established in a previous study (35). Wireless surface EMG and intramuscular wire electrodes were used to analyze the changes in muscle activation and their variability within the same participants and period.

**Figure 1:**
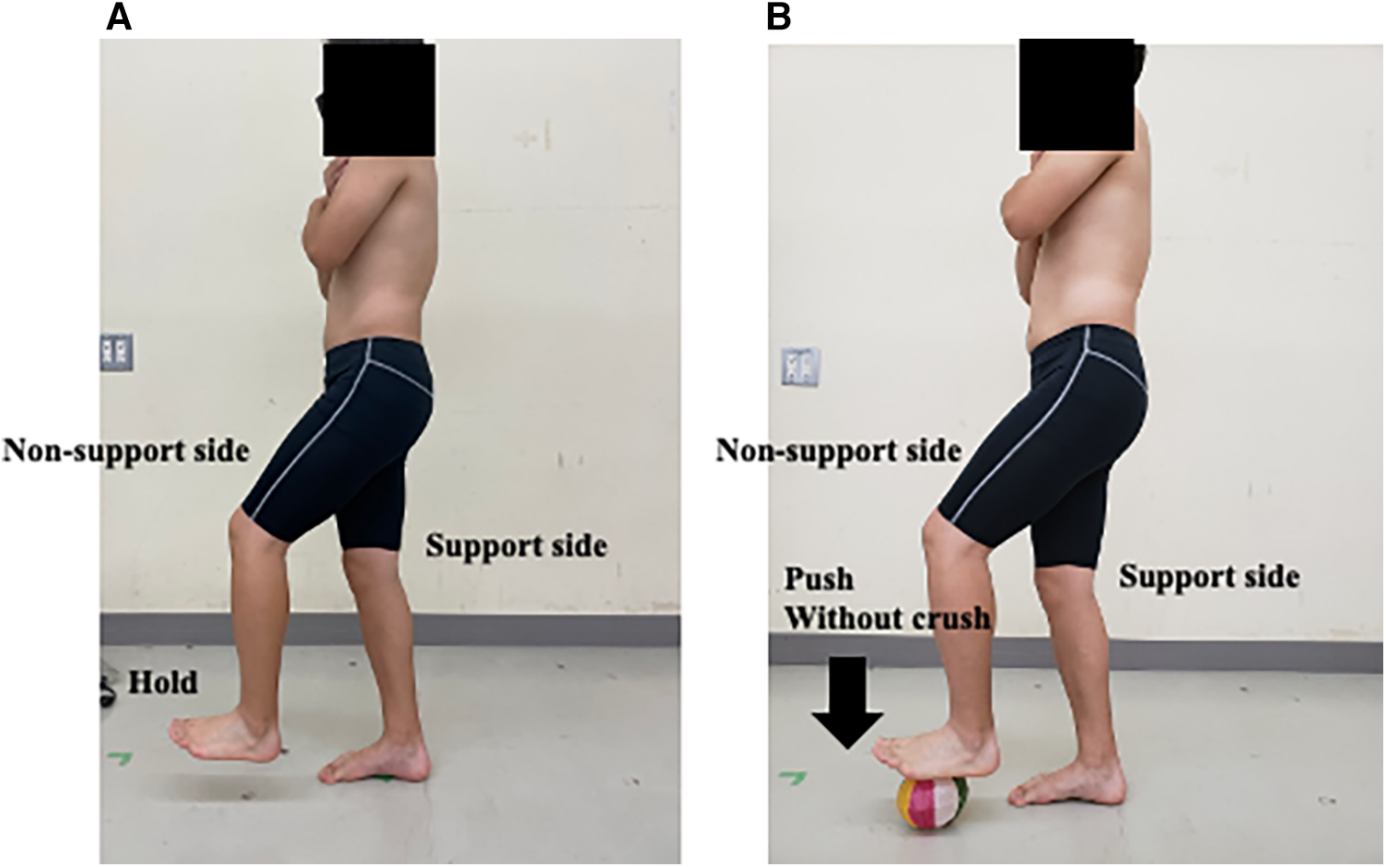
Illustration of the single-leg stance task in each trial on the support and non-support sides. (**A**) Normal single-leg stance, (**B**) external focus instruction using a paper balloon.

Participants were instructed to assume a static single-leg stance position and place the non-supporting foot on the top of the PB and exert high effort by pushing their foot towards the ground for 5 s, while ensuring that the balloon was not crushed. External focus instruction was giving to the participants only in non-support side to control avoid crushing it, and we monitor muscle activation on both support and non-support side (Figure 1). Participants were promptly alerted to instances of the paper balloon being crushed, discernible by the sound of the paper crumpling. Additionally, an examiner monitored the participants' foot position objectively.

Participants maintained the same posture and height on the non-supporting foot for the normal static single-leg stance. They completed two trials for both the right and left sides under the same conditions in random order, a total of four conditions, including left, right, Normal, and PB, were performed three times each. To maintain consistent body posture and joint angles, the examiner continuously evaluated the participants and instructed them to avoid shifting their body weight and to stay centered during the task, which was visually monitored.

All tests were conducted on the same day within one session, and the trials were performed randomly. Each trial was limited to 90 s to prevent potential fatigue from maximum muscle exertion during the exercise task (37). We set at least a one-minute interval between each trial and leg, communicating with the participants between each set of repetitions to ensure they could perform the PB task with maximum effort, incorporating intervals of 10–15 s.

Before coming to the laboratory, none of the participants had prior experience with the external focus instruction using a PB task. However, each participant watched an instructional video before the experiment and had the opportunity to familiarize themselves with the task for 5–10 min before the start of the experiment. The researchers ensured that the PB was properly inflated for each balloon session.

### 2.3 External focus instruction using paper balloon (PB)

Muscle activity can change with different instruction methods and the focus of attention can be classified as either internal focus instruction or external focus instruction (31). External focus instruction directs attention to the intended effects of movements on the environment or external objects, fostering improved performance. In contrast, internal focus instruction involves conscious control of specific muscles or body parts (32, 38). For example, instructing focus on the movement of their wrist (internal focus) during a basketball shot or focusing on the basket (external focus), and generally, research has reported that external focus enhances motor learning performance (33). In this study, we opted for the external focus instruction method, employing a PB as the external object, and executed the task with careful control to prevent object crushing. Furthermore, while external focus instruction has been employed in sports performance previously, there is a need for a more comprehensive examination of its application specifically for exercise.

### 2.4 The paper balloon (PB)

**Figure 2:**
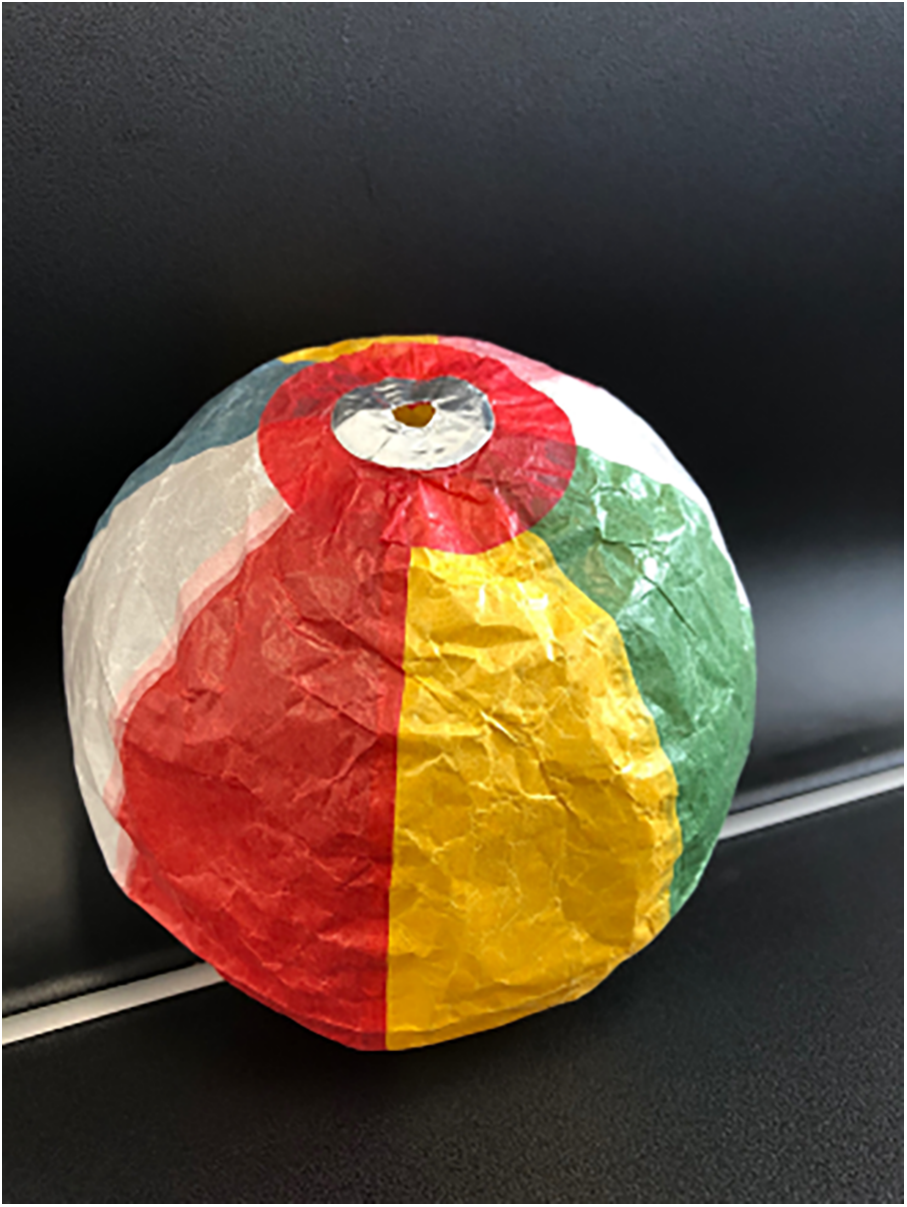
Kamifusen-traditional Japanese paper balloon.

The paper balloon (PB), known as “Kamifusen” in Japanese, is a traditional toy made from rice paper and features a small hole. Remarkably, despite this hole, the PB stays inflated by maintaining proportional air pressure between its interior and exterior. Its soft texture makes it easy to inflate and keep inflated (39) (Figure 2).

### 2.5 EMG

EMG signals were recorded from the following muscles, including the transversus abdominis (TrA), internal oblique (IO), external oblique (EO), rectus abdominis (RA), lumbar multifidus (MF), lumbar erector spinae (LES), gluteus maximus (GMax), gluteus medius (GMed), adductor longus (ADD), rectus femoris (RF), and biceps femoris (BF). EMG data were acquired using a wireless EMG telemeter system (BioLog DL-5000, S&ME Co., Tokyo, Japan) with a sampling rate of 2,000 Hz. The TrA was assessed using bipolar intramuscular fine-wire electrodes (Unique Medical, Tokyo, Japan), whereas the remaining muscles were evaluated using surface electrodes (BlueSensor N-00-S, METS Co., Tokyo, Japan).

**Figure 3:**
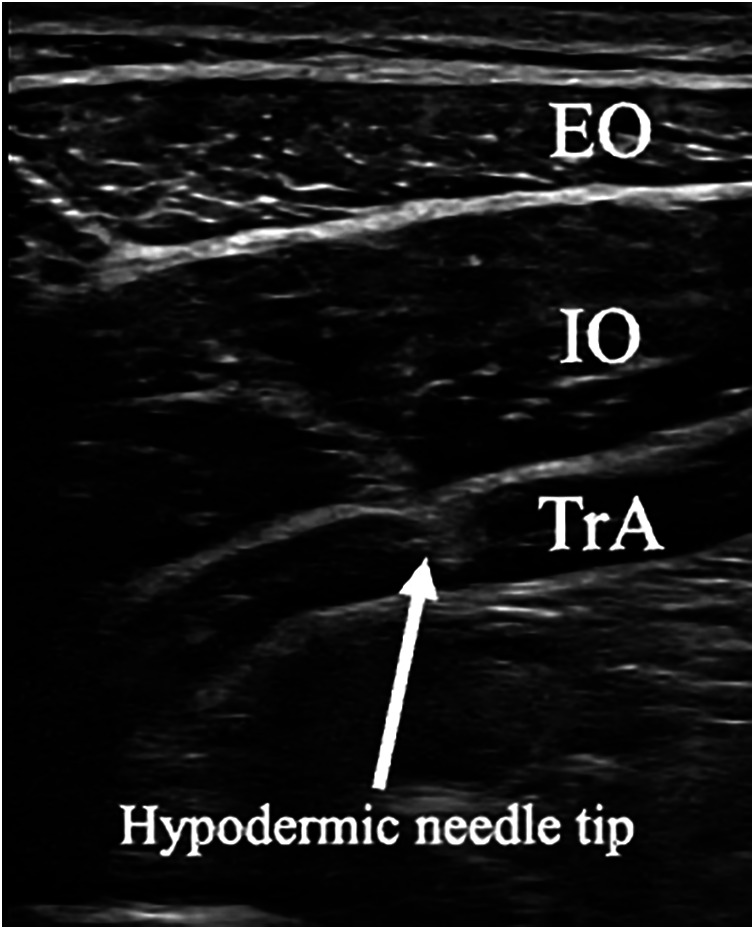
Ultrasound images of the hypodermic needles insertion to transversus abdominis (TrA). EO, external oblique; IO, internal oblique; TrA, transversus abdominis.

The intramuscular fine-wire electrode's 5 mm the outer teflon sheathing diameter, and the 3 mm the inner fine wire diameter were bent to ensure accurate placement to hook the muscles. Except for the tips, the electrodes were coated with Teflon. The electrodes were carefully inserted into the TrA muscle belly at the navel level under ultrasonographic guidance (LOGIQ e; GE, Boston, USA). We thoroughly checked the TrA with ultrasound images and then inserted it at a predetermined depth, correct insertion into the TrA was confirmed by observing the EMG amplitude during a mild abdominal hollowing maneuver, facilitating isolated TrA muscle contraction (40). Correct insertion was defined as an increased muscle activity isolated from IO muscle activity (Figure 3).

The surface electrodes had a diameter of 8 mm, and the distance between them was set to 20 mm. Electrodes were attached to the muscle belly following the Surface ElectroMyoGraphy for the Non-Invasive Assessment of Muscles guidelines. To reduce skin impedance, the participants' hair was shaved, and the skin was cleaned with an abrasive and alcohol.

**Figure 4:**
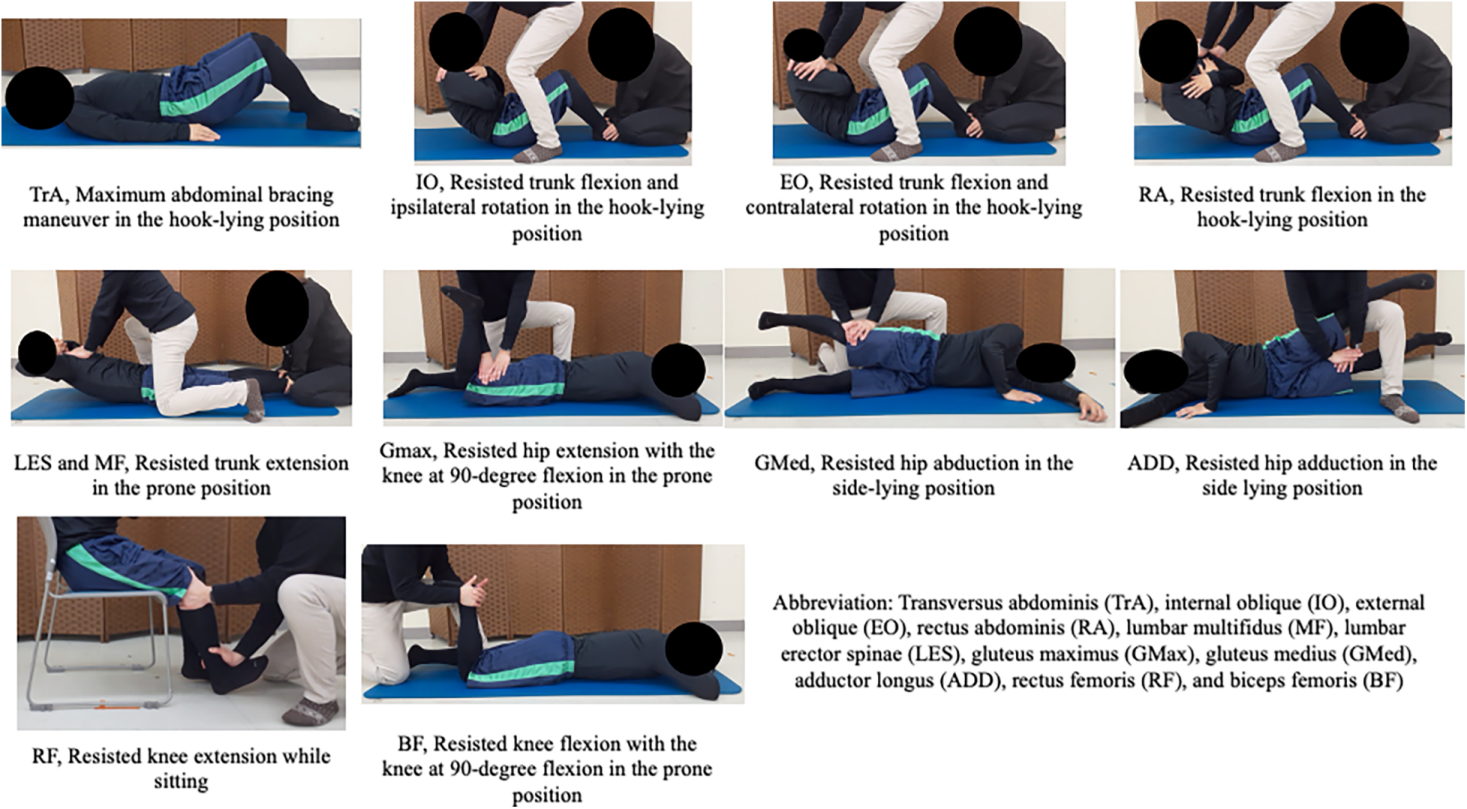
Maximum voluntary isometric contractions (MVICs).

Maximum voluntary isometric contractions (MVICs) were measured in each muscle using the following procedures (Figure 4) (41–47): TrA, maximum abdominal bracing maneuver in the hook-lying position; IO resisted trunk flexion and ipsilateral rotation in the hook-lying position; EO resisted trunk flexion and contralateral rotation in the hook-lying position; RA resisted trunk flexion in the hook-lying position; LES and MF resisted trunk extension in the prone position; GMax resisted hip extension with the knee at 90-degree flexion in the prone position; ADD, resisted hip adduction in the side lying position; GMed resisted hip abduction in the side-lying position; RF, resisted knee extension while sitting; and BF, resisted knee flexion with the knee at 90-degree flexion in the prone position. Each MVIC measurement was performed once for 5 s.

### 2.6 EMG data analysis

EMG data analysis was conducted using BIMUTAS-Video software (KISSEI COMTEC Co., Ltd., Nagano, Japan). The EMG signals were pre-processed to remove motion artifacts and ensure an accurate representation of muscle activity. First, the EMG data were high-pass filtered at 10 Hz and low-pass filtered at 950 Hz to eliminate unwanted noise or motion-related interference. The filtered data were then rectified to ensure that only the absolute values of the EMG signals were used for further analysis. To quantify muscle activation during each task and the MVICs, root mean square (RMS) values were calculated for the middle 3 s of the maximum contraction periods, this approach allows for a representative assessment of muscle activation during tasks and MVICs (48). The RMS values obtained during each task were normalized to the RMS values using the MVICs, this normalization allowed the comparison of muscle activation levels across different tasks and individuals. The normalized RMS values are expressed as percentages of the MVICs and presented as %MVICs. The %MVICs from three trials in each task were averaged to ensure statistical robustness used for subsequent statistical analyses, this EMG data analysis approach allowed for a comprehensive evaluation of muscle activation during the tasks, considering individual variations and providing meaningful insights into the participants' muscle activation patterns.

### 2.7 Statistical analyses

Statistical analyses were performed using IBM SPSS Statistics software version 29.0 (IBM Corp., Armonk, NY, USA). The Shapiro–Wilk test was used to assess the normality of the data distribution. Because most of the data were not normally distributed, two-way analysis of variance was not appropriate. Depending on the normality of the data distribution, the unpaired *t*-test or Mann–Whitney *U*-test was employed to examine differences between the exercise tasks and between the sides.

The alpha level for statistical significance was set at 0.05. Given that the unpaired *t*-test or Mann–Whitney *U*-test was performed four times for each muscle, the significance level was adjusted to 0.0125 (0.05/4) using the Bonferroni correction method.

Data are expressed as median (interquartile range) to account for potential non-normality in the distribution. Cohen's *d* and its 95% confidence interval were calculated to determine the effect size, where values of 0.20–0.49 indicated a small effect; 0.50–0.79, a medium effect; and ≥0.8 a significant effect (49).

### 2.8 Results

**Figure 5:**
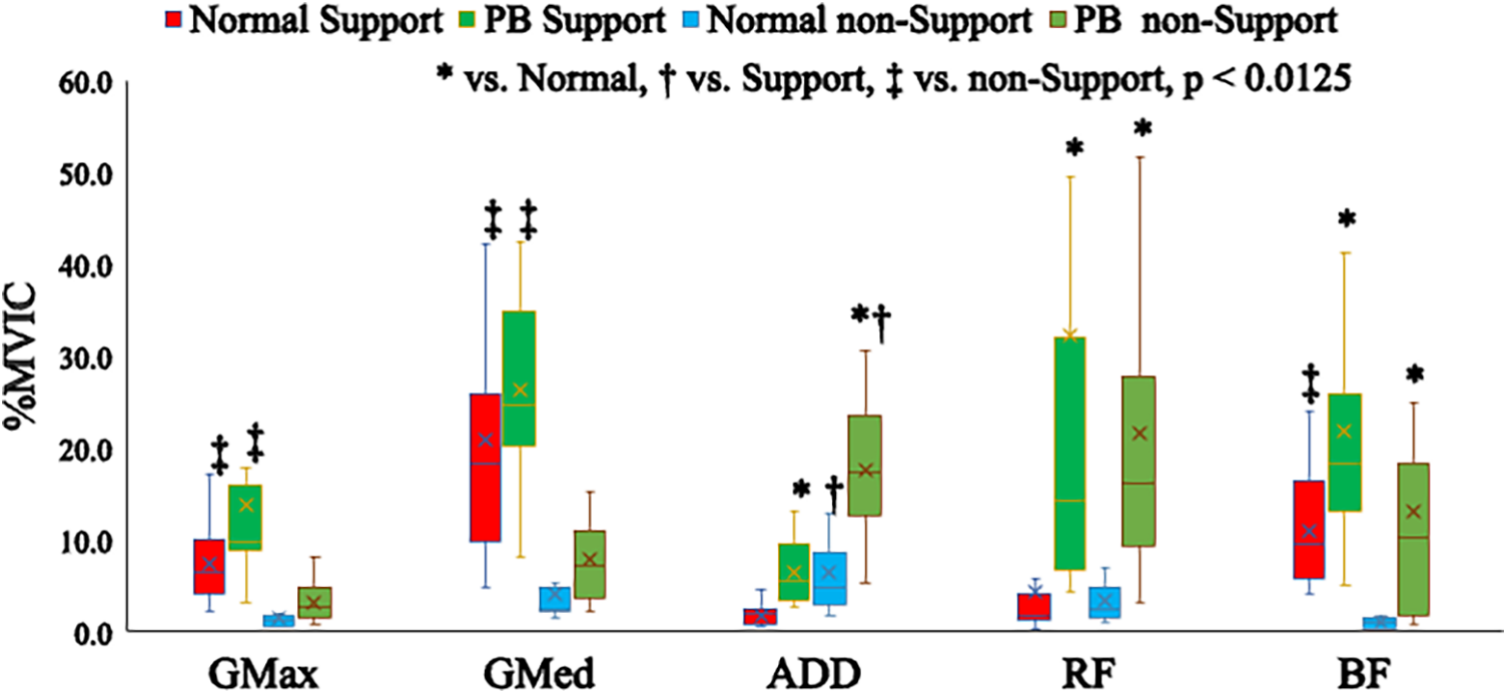
Differences in muscle activity in the trunk muscles between exercise tasks.

**Figure 6:**
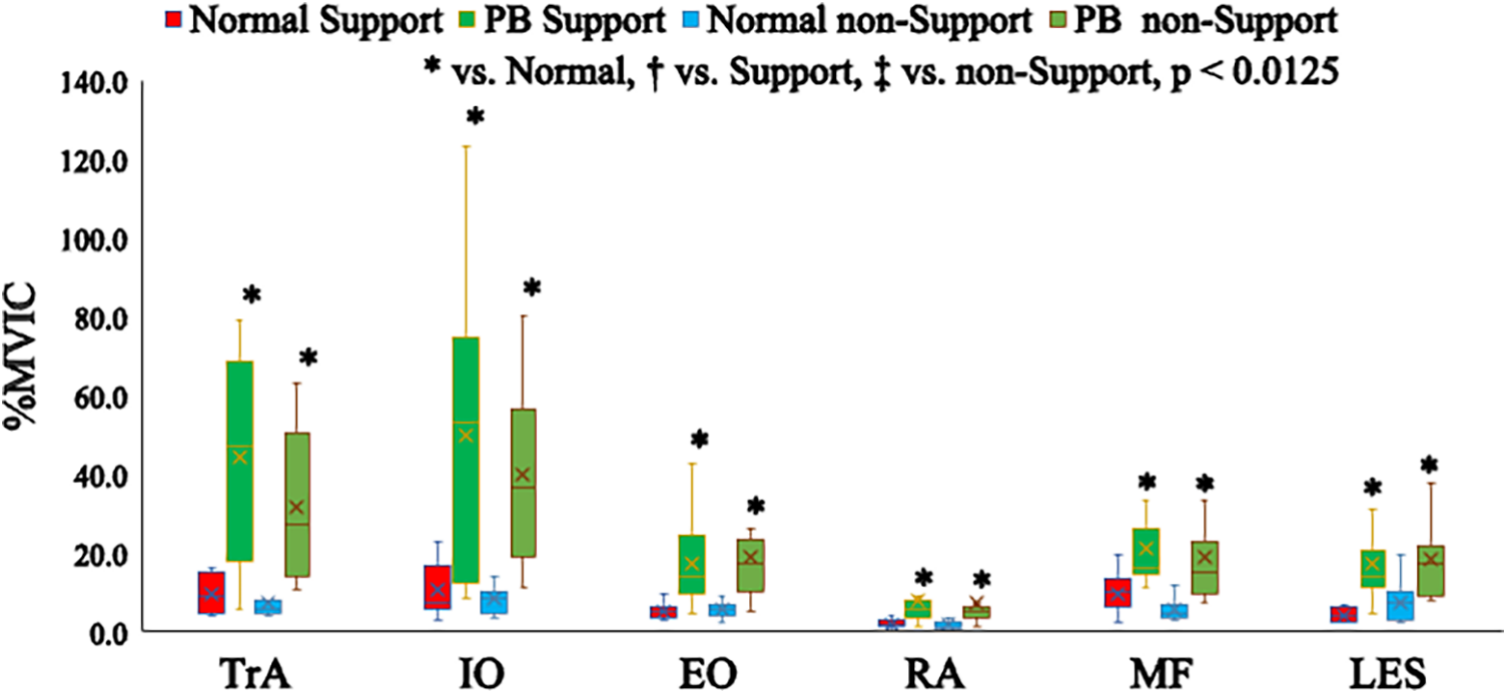
Differences in muscle activity in the lower extremity muscles between exercise tasks.

The medians and interquartile ranges of muscle activity for each exercise task are presented in Figures 5, 6. The results of statistical analysis are shown in Tables 1, 2 and descriptive statistics in Table 3. Regarding exercise task differences, bilateral TrA, IO, EO, RA, MF, LES, ADD, RF and BF activations were significantly higher during the PB task than during the normal single-leg stance.

**Table 1:** Comparison of trunk and lower limb muscle activity between the support and non-support sides for each trial.

		*z*-value *t*-value^a^	*p*-value *p* < 0.0125*	Cohen's *d* (95% confidence interval)
TrA	Support Normal vs. PB	−4.890^a^	<0.001*	−1.918 (−2.777 to −0.938)
	non-Support Normal vs. PB	−3.924	<0.001*	−1.740 (−2.580 to −0.790)
IO	Support Normal vs. PB	−3.104	0.001*	−1.510 (−2.328 to −0.597)
	non-Support Normal vs. PB	−3.976	<0.001*	−1.921 (−2.781 to −0.940)
EO	Support Normal vs. PB	−3.993^a^	0.002*	−1.566 (−2.389 to −0.644)
	non-Support Normal vs. PB	−4.519^a^	0.001*	−1.773 (−2.616 to −0.817)
RA	Support Normal vs. PB	−3.565	<0.001*	−0.854 (−1.628 to −0.026)
	non-Support Normal vs. PB	−3.565	<0.001*	−0.766 (−1.536–0.053)
MF	Support Normal vs. PB	−3.258	0.001*	−1.333 (−2.136 to −0.446)
	non-Support Normal vs. PB	−3.822	<0.001*	−1.873 (−2.728 to −0.901)
LES	Support Normal vs. PB	−4.078	<0.001*	−1.778 (−2.621 to −0.821)
	non-Support Normal vs. PB	−3.104	0.001*	−1.294 (−2.094 to −0.412)
GMax	Support Normal vs. PB	−2.283	0.022	−0.808 (−1.580–0.015)
	non-Support Normal vs. PB	−2.385	0.016	−0.957 (−1.736 to −0.118)
GMed	Support Normal vs. PB	−1.150^a^	0.261	−0.451 (−1.215–0.341)
	non-Support Normal vs. PB	−2.437	0.014	−0.983 (−1.763 to −0.141)
ADD	Support Normal vs. PB	−4.366^a^	0.001*	−1.713 (−2.550 to −0.767)
	non-Support Normal vs. PB	−3.463	<0.001*	−1.755 (−2.597 to −0.803)
RF	Support Normal vs. PB	−3.668	<0.001*	−0.725 (−1.494–0.090)
	non-Support Normal vs. PB	−3.924	<0.001*	−1.501 (−2.318 to −0.590)
BF	Support Normal vs. PB	−2.539	0.010*	−0.974 (−1.754 to −0.133)
	non-Support Normal vs. PB	−3.514	<0.001*	−1.172 (−1.963 to −0.307)

PB, paper balloon; TrA, transversus abdominal; IO, internal oblique; EO, external oblique; RA, rectus abdominal; MF, multifidus; LES, lumber erector spinae; GMax, gluteus maximus; GMed, gluteus medius; ADD, adductor longus; RF, rectus femoris; BF, biceps femoris.

The superscript symbol “a” is used to indicate *t*-value.

**Table 2:** Comparative analysis of trunk and lower extremity muscle activities across trials for each muscle.

		*z*-value *t*-value^a^	*p*-value *p* < 0.0125*	Cohen's *d* (95% confidence interval)
TrA	Normal Support vs. non-Support	1.206	0.243	0.540 (−0.259–1.305)
	PB Support vs. non-Support	1.441^a^	0.162	0.565 (−0.236–1.330)
IO	Normal Support vs. non-Support	0.539	0.614	0.351 (−0.435–1.114)
	PB Support vs. non-Support	0.810^a^	0.426	0.318 (−0.466–1.081)
EO	Normal Support vs. non-Support	−0.394^a^	0.697	−0.155 (−0.920–0.620)
	PB Support vs. non-Support	−0.417^a^	0.680	−0.164 (−0.928–0.612)
RA	Normal Support vs. non-Support	0.627^a^	0.536	0.246 (−0.533–1.010)
	PB Support vs. non-Support	0.846	0.418	0.066 (−0.706–0.832)
MF	Normal Support vs. non-Support	2.283	0.022	1.054 (0.204–1.838)
	PB Support vs. non-Support	0.949	0.362	0.181 (−0.595–0.945)
LES	Normal Support vs. non-Support	−2.174^a^	0.046	−0.853 (−1.626 to −0.025)
	PB Support vs. non-Support	−0.487	0.650	−0.142 (−0.907–0.632)
GMax	Normal Support vs. non-Support	4.181	<0.001*	1.826 (0.862–2.675)
	PB Support vs. non-Support	3.976	<0.001*	1.402 (0.505–2.210)
GMed	Normal Support vs. non-Support	3.873	<0.001*	1.677 (0.737–2.510)
	PB Support vs. non-Support	5.982^a^	<0.001*	2.346 (1.286–3.258)
ADD	Normal Support vs. non-Support	−3.617	<0.001*	−1.256 (−2.053 to −0.380)
	PB Support vs. non-Support	−4.928^a^	<0.001*	−1.933 (−2.794 to −0.950)
RF	Normal Support vs. non-Support	−0.846	0.418	0.187 (−0.589–0.951)
	PB Support vs. non-Support	−0.282	0.801	0.267 (−0.513–1.031)
BF	Normal Support vs. non-Support	4.335	<0.001*	2.059 (1.054–2.935)
	PB Support vs. non-Support	2.078	0.039	0.611 (−0.193–1.377)

PB, paper balloon; TrA, transversus abdominal; IO, internal oblique; EO, external oblique; RA, rectus abdominal; MF, multifidus; LES, lumber erector spinae GMax, gluteus maximus; GMed, gluteus medius; ADD, adductor longus; RF, rectus femoris; BF, biceps femoris.

The superscript symbol “a” is used to indicate *t*-value.

**Table 3:** Descriptive data of trunk and lower limb muscle activity between the support and non-support sides for each trial.

Median (Interquartile range)	Normal support	PB support	Normal non-support	PB non-support
TrA	8.8 (9.3)	47.3 (50.3)	5.8 (3.0)	27.1 (33.6)
IO	7.6 (7.2)	53.4 (53.3)	8.4 (4.3)	36.8 (29.7)
EO	5.2 (1.8)	14.0 (12.5)	5.9 (2.1)	17.6 (11.6)
RA	1.8 (1.6)	6.0 (3.6)	1.6 (1.5)	5.0 (2.6)
MF	10.4 (5.6)	16.1 (8.3)	4.6 (1.8)	15.1 (10.0)
LES	3.9 (3.2)	14.2 (2.7)	7.2 (5.9)	17.2 (8.4)
GMax	6.4 (3.7)	9.9 (5.1)	1.3 (1.1)	2.6 (2.0)
GMed	18.3 (13.4)	24.6 (10.7)	2.6 (2.1)	7.2 (7.1)
ADD	2.0 (1.4)	5.6 (5.7)	4.9 (4.9)	17.3 (9.5)
RF	1.8 (1.3)	14.3 (24.8)	2.4 (2.9)	16.2 (17.7)
BF	9.6 (6.0)	18.3 (12.1)	1.0 (1.1)	10.2 (12.0)

PB, paper balloon; TrA, transversus abdominal; IO, internal oblique; EO, external oblique; RA, rectus abdominal; MF, multifidus; LES, lumber erector spinae; GMax, gluteus maximus; GMed, gluteus medius; ADD, adductor longus; RF, rectus femoris; BF, biceps femoris.

Regarding the differences between the sides, GMax and GMed activation on the supporting side was significantly higher than that on the non-supporting side during both the PB task and normal single-leg stance. However, the BF activation on the supporting side was significantly higher than that on the non-supporting side only during the normal single-leg stance. Interestingly, ADD activation on the non-supporting side was significantly higher than that on the supporting side during both the PB task and normal single-leg stance.

## 3 Discussion

This study aims to explore a novel isometric method employing an external focus instruction with a PB, comparing it with a standard single-leg stance and external focus instruction using a PB in the same position. External focus instruction was giving to the participants only in non-support side to control avoid crushing towards to the ground and we monitor core muscle activation involving the trunk and lower extremities on both support and non-support side. We hypothesized that external focus instruction using a PB condition would result in greater activation compared to the normal condition, specifically in the muscles of the trunk and lower extremities on both the support and non-support sides during a single-leg stance.

The EMG data analysis revealed that the participants exhibited distinct trunk and lower extremity muscle activation profiles using a PB in the external focus instruction task. Particularly, the external focus instruction single leg task had a notable impact on muscle activation levels on the non-supporting side. Moreover, trunk muscle activation during the PB task was significantly higher than that in the normal single-leg stance.

Our study revealed a novel finding: mechanism alters the degree of co-contraction of the antagonistic muscles to avoid crushing the PB task led to increased trunk and lower extremity muscle activities. This is consistent with Prior et al. (50), who reported that trunk muscle activation contributes to the stability of the single-leg stance. Moreover, increased trunk muscle activation holds the spine in a neutral position, can reduce the risk of lower back sports injuries (51, 52), while also playing a crucial role in providing stability and support to the spine (1, 2). This highlights the importance of our study in enhancing trunk muscle activity.

In the lower extremity, the gluteus maximus and hamstrings primarily involve hip extension (53, 54). The gluteal region is crucial for lower limb and pelvic stability and contains essential neurovascular structures—the gluteus medius functions as a hip abductor necessary for locomotion (55). Gluteus medius activity is important for controlling knee and pelvic stability during single leg stance (56). Therefore, the current study showed that exercise with external focus instruction using a PB in single leg stance can activate the gluteal muscles and hamstrings.

Murofushi et al. (34–36) introduced a novel isometric exercise involving an external focus instruction technique using a PB, in which participants exert control to avoid crushing it. This method alters the degree of contraction, leading to trunk enhanced muscle activation in chest squeeze and front plank position. In current study, an external-focus instruction using a PB while exerting control to avoid crushing during a static single-leg stance, pushing the non-supporting foot toward the ground, enhanced core muscle activities in the trunk and lower extremities compared with that of the normal static single-leg stance.

Further for the best of our knowledge, no previous studies have investigated how muscle activity is affected by using an external focus instruction technique with a PB task during a single-leg stance, particularly in terms of monitoring muscle activity on the contralateral side of the body.

This study sheds light on a novel area of inquiry, as no previous studies have explored how muscle activity is affected by employing an external focus instruction technique with a PB task during a single-leg stance, particularly in terms of monitoring muscle activity on the contralateral side of the body. The nature of the neural adaptation during contralateral training must be clarified. However, these mechanisms can potentially improve the effectiveness of resistance training protocols. These cross-limb effects can be leveraged to support the recovery of patients with movement disorders primarily affecting one side of the body (57, 58). The contralateral training phenomenon involves neural adaptations expected at the spinal cord level (59, 60).

Moreover, this study revealed that muscle activities significantly activated the contralateral side muscles on the TrA, IO, EO, MF, LES, ADD, and RF while exerting control to avoid crushing during the single-leg stance push.

### 3.1 Clinical implications

The results of this study offer valuable insights for clinicians, trainers, and researchers.

External-focus instruction with cognitive control using a PB offers the advantage of activating trunk and lower extremity muscles without bearing weight, potentially avoiding pressure on the spine, knee, or hip joints. From the perspective of sports injury prevention, including knee injuries, activating the trunk and gluteal muscles improves dynamic lower extremity alignment during sports activities (61, 62). Therefore, external focus instruction using a PB in the single leg stance is an effective training method.

Additionally, the study revealed that how individuals direct their attention to the non-supporting side influences muscle activation on the supporting side. While our study focused on static standing, its implications extend to dynamic activities like sports movements and walking. Redirecting attention to muscle activity on the non-supporting side might influence muscle activation on the supporting side, impacting overall movement patterns. Although our study primarily captured responses rather than exercise adaptations, these findings suggest potential implications for enhancing movement efficiency and performance in various activities and sports.

### 3.2 Limitations

This study had several limitations. First, the sample size was relatively small owing to the invasive nature of the intramuscular wire electrodes. Second, only male participants were recruited. The study's results could change with different participants, such as female or injured patients. Expanding the participant pool in future studies may strengthen the generalizability of our findings.

Future research can build upon these findings to explore the long-term effects of exercise interventions utilizing external focus instructions and investigate their impact on injury prevention and functional performance in various populations.

## 4 Conclusions

The external focus PB task during single leg stance significantly increased the trunk and lower extremity muscle activation on both the support and non-support sides. These findings suggest that external focus using a PB may be explored as a potential tool for designing exercise programs that involve trunk stability and lower extremity muscles. Further research is needed to establish its effectiveness in injury prevention and enhancing coordination and balance. Incorporating this task into rehabilitation and training protocols may offer benefits for optimizing muscle activation patterns and promoting overall stability during single-leg stance exercises.

## Data Availability

The raw data supporting the conclusions of this article will be made available by the authors, without undue reservation.
